# Case of Inflammation of the Vena Cava, Iliaca, and Femoral Veins

**Published:** 1831-04-01

**Authors:** 


XIII.

STEEVENS' HOSPITAL.

### CASE OF INFLAMMATION OF THE VENA CAVA, ILIAC, AND FEMORAL VEINS. By JOHN CRAMPTON, M.D.

A BOY, aged 14 years, was admitted into Steevens' Hospital, April 1st, 1830, labouring under considerable swelling of the whole abdomen, which was very tense and sore to the touch, with sense of fluctuation. The limbs were very much swollen, apparently to their utmost extent—tender on pressure—and exhibiting large blue veins very conspicuously, both on the limbs and abdomen. The breathing was anxious and disturbed, pulse frequent and hard, occasionally irregular, countenance bloated, with a purple tinge. He could scarcely lie down, and he complained also of distressing palpitation of the heart. His bowels were confined, though he had taken aperient medicines. The urine was scanty and high-coloured. The disease commenced a fortnight before his reception into hospital, with intense inward pain in the epigastric region. Venesection had been attempted, but failed—leeches were then applied, but no relief obtained. The breathing became more embarrassed, and the dropsical symptoms increased.

Stethoscopic examination shewed the lungs to be sound; but the action of the heart was occasionally strong, tumultuous, and irregular—then again quiet. 2d April, the day after admission, he was bled—and took calomel, jalap, and cream of tartar internally. 5th April. Leeches to the chest—and next day to different parts of the abdomen—small doses of elaterium—castor oil and oil of turpentine. 8th. Leechings repeated, and the other parts of treatment continued; but no relief was yet obtained. On the 10th, the right leg and thigh assumed the appearance of phlegmasia dolens, being swollen enormously and very painful on pressure. On the 12th, the limb became erysipelatous, with severe pain in the groin, to which leeches were applied, with fomentations, &c. but the limb proceeded to a gangrenous state, and the boy died on the 17th April. We shall give the dissection in the words of Mr. Bond, who examined the body.

"Skin jaundiced; superficial veins on the anterior part of the chest, dilated and varicose; lower extremities swollen, particularly the right, which was gangrenous in the anterior and upper part of the thigh, and the inferior part of the leg, in its whole circumference. An incision gave exit to a yellow serum, which, with adipose tissue, occupied the thickness of two inches between the skin and muscles.

On opening the abdomen, the cavity contained a pint of citron-coloured fluid, the membranous viscera were pale and free from disease, the stomach contracted and empty, spleen and kidneys natural. The liver was slightly enlarged, its structure of a marbled appearance. The lobulus Spigelii was considerably larger than natural, softened, and of a light yellow colour.

The femoral and iliac veins, as also the cava for three inches and a half above its bifurcation, were contracted, thickened, and partially imbedded in a granular fatty substance, their calibre clogged up by masses of ash-coloured lymph, and in some points wider than the rest, by half-coagulated blood; an adventitious membrane of a rich carmine colour lined the inner coat of the vessel, and from which it could be dissected, leaving this coat sound, but this with the external coat, from which it was easily separated, was white and thickened like an artery. This membrane was in some places disorganized, as if it suffered from gangrene.

A large oval tumor, two and a half inches in length, and one and a half in diameter, filled the vein from its entrance into the auricle, to within an inch of the point where the vein was closed; it was connected by slight adhesions to the lining membrane of the vein, solid, composed of fibrine, disposed in cells, enclosed in a delicate capsule of light yellow colour, with slight traces of vascularity through it; a scrofulous tumor the size of a walnut was connected with the former, and made some pressure on the cava.

The venæ hepaticæ were also blocked up with fibrine. The lungs were healthy; the pleura contained a quantity of fluid similar to that in the abdomen; the pericardium contained about six ounces of fluid; the heart was small, but free from organic disease: the head was not examined."

Dr. C. informs us, that "intense pain in the epigastrium was the first symptom" in this case—and he surmises that the vena cava itself was probably the part first affected with inflammation—and obstructed by the usual deposits of fibrinous concretion—and, finally, that this secretion or perturbed action of the vein extended downwards to the iliacs, on arrival at which, "the usual symptoms of phlegmasia dolens" ensued. Now we would ask Dr. Crampton, or any other practitioner of experience, whether he believes this to be the usual state of things in the last-mentioned disease, not one case out of a hundred of which ever proves fatal?

We cannot imagine, indeed, how any one who has seen the phlegmasia dolens of parturient women, can confound it with this serious—generally mortal phlebitis.

"That general hydropic effusion should be the consequence of such a degree of obstruction, or that tumultuous and disturbed action of the heart should attend inflammation of the vena cava, is not surprising. Such sympathetic occurrences are easily explained in those cases where the gradual advancement of the early symptoms of disease, and the order in which they proceed, can be ascertained. A superficial examination might induce a practitioner to consider the heart as the organ primarily concerned, but here the value of the stethoscope was evinced. The action of the heart was not of that character which denotes change of structure in that important organ; no doubt disorder in its function was quite obvious; while the perfect state of integrity of the lungs, although the respiration was embarrassed, intimated that the thoracic organs were not those which were primarily instrumental in the production of the dropsical affection, however they might in the end have conspired towards it by their sympathetic state of disturbance. That a scrofulous diathesis assisted as a predisposition, and as aiding the action of the occasional causes, I believe there can be little doubt. In the series of morbid changes, in this instance, some might suppose the external tumors to have been consecutive to the internal irritation of the coats of the veins, instead of having existed as antecedents, but this is a matter of but little consequence."

Dr. C. thinks it possible that many of those refractory cases of dropsy, which refuse to yield to treatment, may be connected with venous inflammation and its consequences, "as in the ordinary occurrence so often met with in phlegmasia dolens." In opposition to Dr. Crampton, we maintain that the swelling in phlegmasia dolens is not dropsical. It is elastic, and the dent made by the finger rises rapidly again to the common level—so very different is it from dropsy or œdema. It is astonishing that so obvious a distinction should have escaped such a man as Dr. Crampton! We do not doubt that, in his *post-mortem* examinations of what he terms PHLEG. DOLENS, he found the "veins inflamed, and the vessels often plugged up with fibrine and purulent effusion"—because we verily believe that he never examined a case of the disease in question—unless the patient died of some other complaint. We have never seen death result from this disease, nor have we met with any of our brethren who has seen it terminate fatally. That phlebitis is a much more common disease than was imagined some years ago, we readily grant—and as it is very generally fatal, we separate it from phlegmasia dolens.

